# Residual effects of low dose of suvorexant, zolpidem, and ramelteon in healthy elderly subjects: A randomized double‐blind study

**DOI:** 10.1002/npr2.12262

**Published:** 2022-06-24

**Authors:** Sachiko Ito Uemura, Aya Imanishi, Yoshino Terui, Insung Park, Masahiro Satake, GoEun Han, Takanobu Shioya, Takashi Kanbayashi, Seiji Nishino

**Affiliations:** ^1^ Department of Physical Therapy Akita University Graduate School of Health Sciences Akita Japan; ^2^ Department of Psychiatry Akita University Graduate School of Medicine Akita Japan; ^3^ International Institute for Integrative Sleep Medicine (WPI‐IIIS) University of Tsukuba Tsukuba Japan; ^4^ Nikoniko‐en, Long‐Term Care Health Facilities Akita Japan; ^5^ Ibaraki Prefectural Medical Center of Psychiatry Kasama Japan; ^6^ Sleep & Circadian Neurobiology Laboratory, Stanford Sleep Research Center Stanford University School of Medicine Palo Alto California USA

**Keywords:** hypnotics, residual effects, sleep, suvorexant, zolpidem

## Abstract

**Introduction:**

Current hypnotic agents have next‐day residual effects. The new orexin antagonist, suvorexant, has little muscle relaxation effect on the physical and cognitive function in the following morning and daytime. In this study, the effects of suvorexant, zolpidem, ramelteon and placebo in elderly subjects were evaluated.

**Methods:**

Six men and eight women aged 63–75 years received a single tablet and lights were then turned off. Subjects were instructed to sleep from 23:00–6:00 with an interruption from 4:00–4:30 for evaluations. Suvorexant 10 mg, zolpidem 5 mg, ramelteon 4 mg or placebo was administered single time in a randomized, double‐blind and crossover design with a one‐week drug holiday in between each drug. Measures of objective parameters and subjective ratings were obtained every 2 h from 4:00 to 16:00.

**Result:**

No subjects showed serious side effects from physical observations and vital sign checks before and after hypnotics were taken. During the first sleep period, the REM sleep time with suvorexant was especially longer than that with zolpidem. During the second sleep period, suvorexant had shorter sleep latency and longer stage2 sleep time than ramelteon and zolpidem, respectively. During the whole entire sleep, the REM sleep time with suvorexant was longer than zolpidem and placebo. For the body sway test with closed eye, the main effects of the medicines and zolpidem were significantly better than suvorexant and ramelteon.

**Conclusion:**

The changes of physical and cognitive functions in healthy elderly after taking hypnotics were not remarkable. Therefore, these three hypnotics maybe appropriate for the elderly people with insomnia for single‐time low dose administration.

## 1 INTRODUCTION

It has been reported that the prevalence of insomnia among the adults in Japan is 17.3%‐21.5%, of which the frequency of use of hypnotics is 3.5%‐5.4%.^1^, ^2^, ^3^ It has been pointed out that the use of hypnotics is associated with falls^4^, ^5^, ^6^, ^7^ and cognitive impairments^8^ of the elderly. Under these circumstances, appropriate guidelines were provided recently on the use of hypnotics for the elderly in Japan.^9^

The GABAa agonists, benzodiazepines and non‐benzodiazepines (e.g., zolpidem), have a muscle‐relaxing action and are therefore considered to have a high risk of falls.^10^, ^11^, ^12^ The above Japanese guidelines currently recommend the melatonin agonist, ramelteon, and the orexin antagonist, suvorexant.^9^ Ramelteon has the advantage of little muscle relaxant effect.^13^, ^14^ Suvorexant has a long half‐life (10 h) with little muscle‐relaxing action,^15^, ^16^ and is considered to have a low risk of falls due to muscle relaxation during mid‐sleep awakening. It is also considered to be advantageous for sleep disturbances such as wake after sleep onset (WASO) and early morning wakefulness, which are common among elderly people.

Several studies have examined the motor and cognitive functions of the elderly after taking hypnotics.^17^, ^18^, ^19^, ^20^ Furthermore, few studies have been conducted on the psychomotor function at night or in the early morning when the fall is likely to occur due to the residual effect of hypnotics.^21^, ^22^

Previously, we conducted research on a group of GABAa agonists, zolpidem, eszopiclone, triazolam, rilmazafone, zaleplon and the melatonin agonist, ramelteon (not published yet).^22^, ^23^, ^24^, ^25^, ^26^ As a result, single use of these hypnotics was useful for young and elderly subjects.^22^, ^23^, ^24^, ^25^, ^26^

In this study, we conducted an experiment using EEG, and physical and cognitive functions tests with the recommended hypnotics with a weak muscle relaxant effect. Then, we investigated hypnotics for safety in the elderly subjects using melatonin agonist ramelteon, orexin antagonist suvorexant and GABAa agonist zolpidem.

## 2 METHODS

### 2.1 Design

**FIGURE 1:**
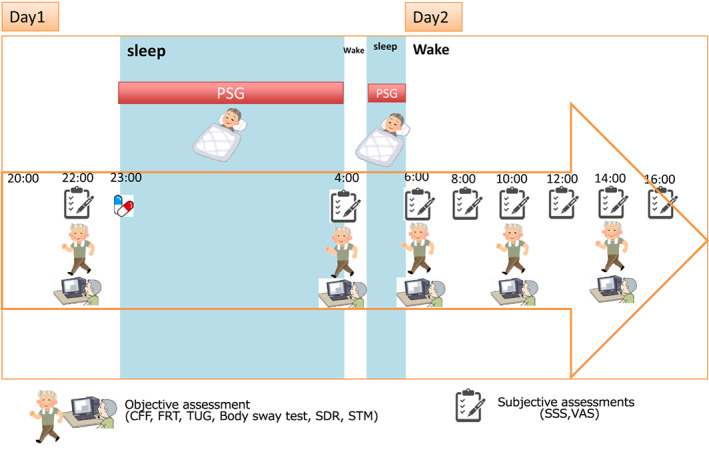
Overview of protocol subjects were randomized to treatment sequences including zolpidem 5 mg, suvorexant 10 mg, and ramelteon 4 mg, and placebo administered in a crossover fashion, with a 6‐day washout period between each treatment. Hypnotics or placebo were orally administered to each subject at bedtime (23:00) and lights were then turned off. On the morning following the administration, lights were turned on at 04:00 and 06:00 (5 and 7 h post medication). Measurement of objective parameters and subjective parameters were obtained beginning at 22:00 (1 h pre‐medication) on Day 1. Subjects remained in the recording room from early in the evening of administration until 17 h post‐dose. All subjects took the same breakfast. The experiment was conducted in a local hotel room. The bedroom temperature was 23°C, the humidity was 38%, and the illumination was 200–300 lux before bedtime. Subjects were instructed to sleep from 23:00 to 6:00 with interruption from 4:00 to 4:30. We prepared the same sleepwear and bedding for them every evening. Dynamic and static balance indexes, objective assessment (CFF, FRT, TUG, body sway test, SDR, STM) and subjective (SSS, VAS) evaluations were performed. Please refer to the method section for details

We conducted a single‐blind pharmacokinetic assessment of 5 mg zolpidem (half‐life: 2.6 h), 10 mg suvorexant (12 h) and 4 mg ramelteon (1‐2.5 h)^27^ in the same subjects (Figure 1) in a randomized, double‐blinded, active‐ and placebo‐controlled manner for 4 weeks. We were approved by Akita University Ethics Committee. This study was carried out in accordance with the principles based on the Declaration of Helsinki, and written informed consent was obtained from all subjects.

### 2.2 Subjects

We recruited healthy 60–75 years old Japanese subjects who fell asleep between 20:00 and 24:00 (Table 1), confirmed by their physical examination, medical history and hematology, and clinical biochemical tests. Any subject who took hypnotics within the previous year are addicted to drugs or alcohol or had a history of repeated falls or a fracture within the past two years was excluded from our study. In total, six men and eight women were enrolled following the inclusion and exclusion criteria. The subjects were advised to refrain from strenuous or unaccustomed exercise during the day.

**TABLE 1:** Demographic date of subjects

List	Average ± SD
age	68.0 ± 3.4 years old (63 ‐75 years old)
gender	6 Males, 8 Females
Body Mass Index (BMI)	22.65 ± 2.6 kg/m^2^
high	158.7 ± 10.3 ㎝
Weight	57.2 ± 9.4㎏
medication	Antihypertensive(6) ∙ Analgesic(2) ∙ No medication(6)
Regular bedtime	11:37 pm
Regular awakening hours	5:51 am
Difficulty getting to sleep	Yes(4) ∙ No(10)
Arousal during sleep	Yes(2) ∙ No(12)
Early morning awaking	Yes(1)
Sleep quality	Good(2) ∙ Not bad (4) ∙ No response (8)
Well‐being	Good(5) ∙ Fair(3) ∙ not bad(1) ∙ No response (5)

We advised the subjects to refrain from prescribed and non‐prescribed medications and supplements. They were also advised to limit their alcohol consumption to two glasses of wine or two small bottles of beer per day. They abstained from alcoholic drinks two days before the admission to the unit or before prestudy and poststudy visit. Caffeine and nicotine were prohibited 1 day before each visit. Four subjects had difficulty falling asleep, two woke up during sleep and one woke up early among 14 subjects.

### 2.3 Procedure

The medication sequences were randomized when administered to the subjects; however, the amounts were kept the same: suvorexant 10 mg, ramelteon 4 mg and placebo with a 6‐day washout period in between each medication. The subjects took the hypnotics or placebo at bedtime (23:00) and lights were then turned off. In the morning following the administration, lights were turned on at 4:00 and 6:00 (5 and 7 h post administration) (Figure 1). On Day 1, we measured the objective parameters and subjective parameters 1 h before the medication at 22:00. The subjects stayed in a local hotel room, from early evening until 17 h after the administration. They all ate the same breakfast. The bedroom temperature was kept at 23°C; the humidity 38%; and the illumination 200–300 Lux before bedtime. Subjects were instructed to sleep from23:00 to 6:00 with an interruption from 4:00 to 4:30. We prepared the same sleepwear and bedding for them every evening.

### 2.4 Sleep evaluation

Before the experiments, we evaluated their sleep based on the Japanese version of the Pittsburgh Sleep Quality Index (PSQI‐J)^28^, ^29^ and confirmed that subjects had no sleep disorders. We also evaluated their sleep schedule cycle and chose the intermediate type, based on the Morningness‐Eveningness Questionnaire (MEQ)^30^ which subjects had completed in advance.

**FIGURE 2:**
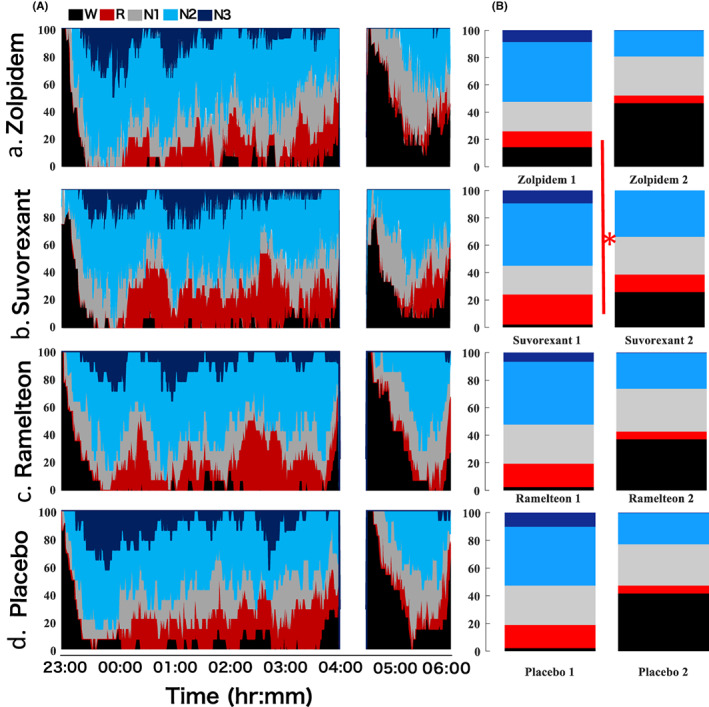
(A) Cumulative display of sleep architecture in all 14 subjects. The percentage of subjects in each sleep stage is shown. Wake (W) is indiated in black; REM (R), red; stage 1 (N1), gray; stage 2 (N2), light blue; and stage 3 (N3), dark blue. We instructed the subjects to go to bed at 23:00 after taking the medication. Then, the subjects were forced awake at 04:00 for various tests, allowed to go back to sleep at 04:30 and finally woken up at 6:00. (B) Percentage of sleep stages in both sleep periods. Suvorexant had longer REM sleep time during 1st periods (23:00‐4:00, p=0.001, indicated in red bar) than zolpidem

All patients underwent an overnight polysomnography (PSG) monitoring on experimental nights (Figure 2), which was obtained using a single‐channel electroencephalogram (EEG) (MOOMIN‐KEI; Sleep Well Co., Osaka, Japan,) as previously described.^31^ According to protocol,^32^ the recorded nights were divided into 30s sequential periods and manually classified into rapid eye movement (REM) sleep and non‐REM sleep, which was further classified into light sleep (N1, N2) or slow wave sleep (N3). Total sleep time (TST) was calculated as the total sleep period minus the wake time after sleep onset (WASO). Sleep onset was defined by the first occurrence of stage 2 sleep, which was followed by 5 min of continuous sleep composed of stage 1, 2, 3, or REM sleep.

### 2.5 Objective assessments

Dynamic and static balance indexes, cognitive and subjective evaluations were performed. It has been confirmed that the dynamic balance indicators TUG and FRT reflect falls in the elderly.^33^, ^34^ The following results are depicted in the supplementary figures S2 and S3.

[Correction added on 12 August 2022, after first online publication: In the objective assessments section, the text were corrected from “This is depicted to Fig. 4” to “The following results are depicted in the supplementary Figures S2 and S3”.]

#### 2.5.1 The timed up and go test (TUG test)

The TUG test was performed according to the method described by Podsiadlo and Richardson.^34^ Participants had to stand up from a sitting position (height of chair = 40 cm) and walk 3 m along a line, perform a 180° turn, and walk back to the chair and sit down; this was timed. The TUG tests were conducted twice, and the best time (where appropriate) was used. Smaller values were better results for TUG.

#### 2.5.2 Functional reach test (FRT)

In the FRT, the protocol described by Duncan et al.^33^ was applied and a GB‐200R (OG giken) was used. Each participant was positioned with one arm raised at 90° and fingers extended. A yardstick was mounted on the wall at shoulder height.

The distance that a participant could reach while extending forward from the initial upright posture to the maximal anterior leaning posture without moving or lifting the feet was visually measured in centimeters (cm), according to where the middle fingertip was positioned on the mounted yardstick. The distances were measured for two attempts, and these were averaged to obtain the FR score. Larger values were better results for FRT.

#### 2.5.3 Body sway test

Body sway test (cm) reflects the standing balance using a stabilometric platform [Zebris WinFDM system (platform), Zebris Medical GmbH, Isny imAllgau, Germany; Foot Print for WindowsR (software), Inter Reha, Tokyo, Japan]. Subjects stood on the platform for 30 seconds (s) in bare feet and with their vision fixed at a point 2 m in front of them at eye level (eyes open) or with their eyes closed.

The sum of the tracks of the center of gravity is the body sway test; the extent of movement of the center of pressure directly relates to the subject’s ability to maintain static balance. For the body sway test, smaller values were better in terms of functionality of the subject.

#### 2.5.4 Critical flicker fusion test (CFF)

This test is believed to assess the integrative capacity of the central nervous system (CNS) under the influence of psychoactive compounds. Subjects were required to discriminate flicker from fusion, and vice versa, of four light‐emitting diodes arranged in a 1 cm square on a black background. Individual thresholds were determined by the psychophysical method of limits on two ascending (flicker to fusion) and two descending (fusion to flicker) scales.^35^ The mean of these two ascending and two descending presentations was used as the threshold frequency in Hz. A decreased threshold was indicative of impairment.

#### 2.5.5 Simple discrimination reaction (SDR) test

The SDR test is included in a performance test software program (Human Response Checker, NoruPro Light Systems, Inc., Tokyo, Japan) to measure the reaction time and hand–eye coordination skills of the subjects.

Subjects were required to right click on a mouse when a blue circle was lit, or left click when a white circle was lit, as quickly as possible. The mean total reaction time (s) and the rate of correct answer (%) of 60 trials were recorded. An increase (slowing) in reaction time was indicative of impairment. The test has been shown to be sensitive to psychoactive compounds. Smaller values were better, in terms of functionality of the subject for SDR reaction time, while larger values were better for SDR accuracy rate (%).

#### 2.5.6 Short‐term memory (STM) test

The STM test has been shown to reflect the retention of short‐term memory. Prior to the study, the subjects underwent an extensive training session to preclude learning effects.

This test is included in the performance test software program: Human Response Checker (NoruPro Light Systems, Inc.). Subjects were required to click the right mouse button when the same number that was displayed three times before the current one appeared or click the left mouse button when a different number appeared. The rate (%) of correct answers in 60 trials was recorded. A decrease in correct answers was indicative of impairment.

### 2.6 Subjective assessments

#### 2.6.1 Stanford sleepiness scale (SSS) and visual analog scale (VAS)

The subjects' sleepiness was evaluated using the SSS.^36^ Alertness, well‐being, and fatigue were evaluated with a VAS at 22:00 (Day 1) and every 2 h from 4:00 to 16:00 (i.e., 4:00, 6:00, 8:00, 10:00, 12:00, 14:00, 16:00) (Day 2). The scale’s extremes were “very drowsy– very alert,” “very bad–very good,” and “very tired–very rested.” In terms of functionality of the subject for the SSS, smaller values were better, while larger values were better for the VAS.

### 2.7 Safety

Physical examinations, vital sign measurements, laboratory tests to ensure safety, and 12‐lead electrocardiograms (ECGs) were performed prestudy and poststudy. In addition, laboratory tests to ensure safety were performed prior to dosing, and vital signs were measured during each treatment period. Subjects were monitored for adverse experiences throughout the study. For each adverse event, the investigator indicated whether or not they thought the event was drug related. This determination was made while the investigator was blinded to the treatment.

### 2.8 Statistical methods

For statistical analysis, a two‐way ANOVA was used, with hypnotics and time as factors. After checking for interaction, a multiple comparison was performed using Bonferroni for the main effects of the medicines or times. Since SDR and sleep evaluations scores were not normally distributed, non‐parametric analysis (Freedman) and a post hoc test (Steel‐Dwass) were used for the statistical calculation. The statistical significance level was *P* < 0.05.

## 3 RESULTS

Throughout this study, no subjects showed serious side effects from physical observations and vital sign checks performed before and after hypnotics were taken.

Cumulative display of sleep architecture in all 14 subjects is presented (Figure 2). The percentage of subjects in each sleep stage is shown. We instructed the subjects to go to bed at 23:00 after taking the medications. Then, the subjects were forced awake at 04:00 for various tests, allowed to go back to sleep at 04:30 and finally woken up at 6:00.

**FIGURE 3:**
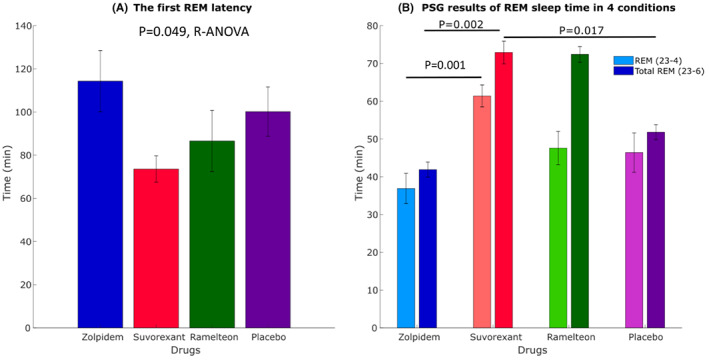
PSG results of REM sleep in 4 conditions, (A) for the REM Latency (23:00‐4:00), the main effects of all the medicines (*P* = 0.049) were seen. (B) The REM sleep time with suvorexant were especially longer than that with zolpidem (23:00‐4:00, *P* = 0.001). The main effects of all the medicines (*P* = 0.001) were seen (23:00‐6:00). The REM sleep time (min) with suvorexant was longer than those with zolpidem and placebo using post hoc test (*P* = 0.002, *P* = 0.017, respectively)

During 23:00–4:00 period, the main effects of medicines were seen for the REM latency (*P* = 0.049) with no significant differences between each compound (Table 2; Figure 3A). For the REM sleep time, the main effects of all the medicines (*P* = 0.001) were seen. The REM sleep time with suvorexant were especially longer than that with zolpidem (*P* = 0.001) (Table 2; Figure 2B; Figure 3B). In 23:00–6:00 periods, the REM sleep time with suvorexant were especially longer than that with zolpidem and placebo (*P* = 0.002, *P* = 0.017, respectively, Figure 3B; Table S1).

**TABLE 2:** EEG results (23:00–4:00)

		Zolpidem		Suvorexant		Ramelteon		Placebo		R‐ANOVA / freedman test, *P*	Post‐hoc test
		Mean	SEM	Mean	SEM	Mean	SEM	Mean	SEM		
TST	(m)	269.8	6.8	274.6	4.9	273.2	4.2	270.3	5.5	ns	
SL	(m)	13.0	1.8	14.8	2.2	14.8	2.8	13.3	2.2	ns	
REML	(m)	114.3	14.2	73.6	6.1	86.6	14.2	100.2	11.4	0.049	ns
WASOT	(time)	4.5	0.7	5.8	0.8	6.6	1.2	5.9	1.2	ns	
WASO	(m)	5.6	1.4	8.6	2.6	9.9	2.1	13.8	3.6	ns	
SE	(%)	89.2	2.2	90.0	1.4	89.7	1.3	88.4	1.8	ns	
REM	(m)	36.9	4.0	61.4	2.9	47.6	4.4	46.4	5.2	0.001	Z < S (0.001)
N1	(m)	68.1	7.9	59.3	5.8	79.3	9.9	78.4	11.4	ns	
N2	(m)	137.8	7.0	127.8	6.2	127.7	6.1	117.3	7.8	ns	
N3	(m)	27.0	6.7	26.1	6.1	18.6	5.0	28.1	6.7	0.006	ns

For the stage3 NREM sleep, the main effects of medicines were found (*P* = 0.006); however, the significant differences were not found between each compound (Table 2; Figure 2B; Figure S1A). The time in bed, total sleep time, sleep latency, and sleep efficiency were not significantly different among all the conditions from 23:00 to 4:00 (Table 2).

**FIGURE 4:**
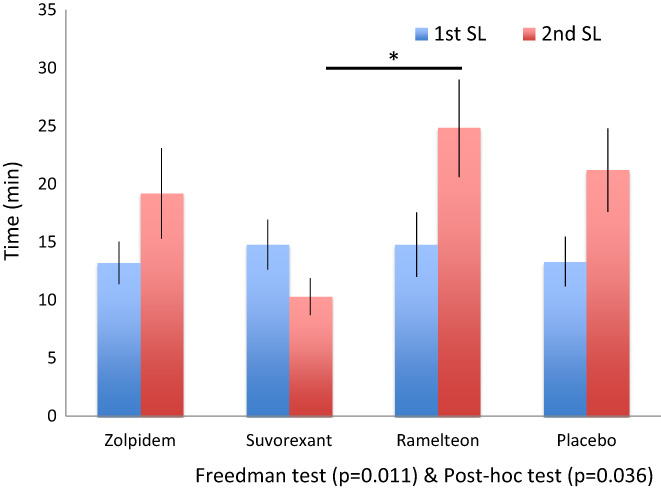
Comparisons of sleep latencies (SL) between the first and the second sleep periods The main effects of medicines were not seen for the first SL. The main effects of medicines were seen for 2nd SL (*P* = 0.011), and the 2nd SL of suvorexant was significantly shorter than that of ramelteon with post hoc test (*P* = 0.036). [Correction added on 12 August 2022, after first online publication: The last sentence for Figure 4 legend has been amended and was originally ‘The main effects of medicines were seen for 2nd SL (0.011), suvorexant was significantly longer than ramelteon with post hoc test (*P* = 0.036)”.]

During 4:30–6:00 period, the main effects of medicines were seen for TST (*P* = 0.039), with no significant differences between each compound (Table 3; Figure 2B). The main effects of medicines were seen for SL (*P* = 0.011), SL of suvorexant was significantly shorter than of ramelteon with post hoc test (*P* = 0.036, Table 3; Figure 4). The main effects of medicines were seen for the N2 sleep stage time (*P* = 0.035), with no significant differences between each compound (Table 3; Figure 2B; Figure S1B).

**TABLE 3:** EEG results (4:30–6:00)

		Zolpidem		Suvorexant		Ramelteon		Placebo		Freedman test / R‐ANOVA, *P*	Post‐hoc test
		Mean	SEM	Mean	SEM	Mean	SEM	Mean	SEM		
TST	(m)	48.0	5.9	66.8	4.6	56.6	4.8	52.5	5.8	0.039	ns
SL	(m)	19.2	3.9	10.3	1.6	24.8	4.2	21.2	3.6	0.011	S < R (0.036)
REM	(m)	5.0	2.0	11.5	3.0	5.0	2.1	5.1	2.0	ns	ns
N1	(m)	25.8	4.0	24.8	1.6	28.0	2.8	26.8	3.3	ns	ns
N2	(m)	17.1	4.2	30.5	2.5	23.5	3.4	20.3	3.6	0.035	ns (Z<S; 0.065)

*Notes*: Mean time of each sleep stage in 14 subjects. The time of subjects in each sleep stage is shown. The subjects were forced awake at 04:00 and went back to sleep at around 04:30. During this 30 min period, the subjects took objective and subjective examinations. TST: The main effects of medicines were seen for TST, with no significant differences between each compound (*P* = 0.039). Stage 2 sleep: The main effects of medicines were seen for stage2 sleep time (*P* = 0.035). Suvorexant was marginally longer than zolpidem with post hoc test (*P* = 0.065).

**FIGURE 5:**
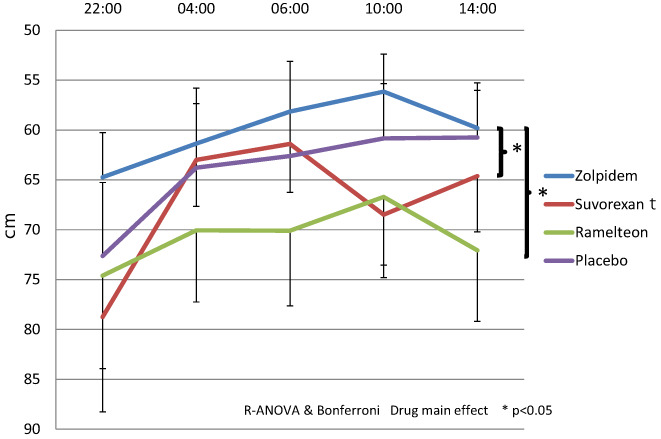
Objective assessment: Body sway test (eyes closed) For the body sway test (closed eyes), we found the main effects of the medicine (*P* = 0.012), which showed body sway movements after zolpidem administration were significantly less than after suvorexant and ramelteon administrations

As for the objective indexes, the main effect in time was observed from all the hypnotics; especially at 4:00 and 6:00, the time main effects were observed in TUG (*P* = 0.002), Body sway test Eyes open (P=0.002),CFF (*P* = 0.0001), SDR‐time (*P* = 0.01) and STM (*P* = 0.02) examination without variance among the medications (Table 4; Figure S3). For the body sway test (closed eyes), we found the main effects of the medicine (*P* = 0.012) (Table 4; Figure 5), which showed body sway movements after zolpidem administration were significantly less than after suvorexant and ramelteon administrations (*P* = 0.03, *P =* 0.04). The other parameters showed no significant differences from the main effects of the medicine (Table 4; Figures S2 and S3).

**TABLE 4:** Objective assessments

		Zolpidem		Suvorexant		Ramelteon		Placebo		Drug Main effect, *P*	Drug Post hoc test	Time Main effect	Time Post hoc test
		Mean	SEM	Mean	SEM	Mean	SEM	Mean	SEM				
TUG	(sec)	4.7	0.2	4.7	0.2	4.6	0.2	4.6	0.2	ns		0.002	4>6* 4>10* 4>14*
FRT	(cm)	42.1	1.2	41.9	1.3	42.3	1.4	41.4	1.4	ns		ns	
Body sway test Eyes open	(cm)	42.5	2.4	45.8	3.0	46.6	3.3	45.6	3.3	ns		0.002	4>6*
**Body sway test Eyes closed**	**(cm)**	**60.0**	**4.6**	**67.3**	**6.5**	**70.7**	**7.5**	**64.1**	**5.8**	**0.012**	* **Z<S** * **Z<R**	**ns**	
CFF	(Hz)	32.7	0.6	32.7	0.7	32.2	0.5	32.6	0.6	ns		0.000	22>6* 22>10** 22>14*
SDR	(%)	92.6	2.1	92.7	1.9	92.7	2.5	91.8	2.5	ns		ns	
	(s)	0.50	0.01	0.50	0.01	0.50	0.01	0.49	0.01	ns		0.01	ns
STM	(%)	72.2	3.4	76.1	3.7	73.5	3.9	74.1	3.8	ns		0.02	ns

*Note*: R‐2way ANOVA & Bonferroni.

*P < 0.05

**P < 0.01

As for the subjective indexes, the main effect in time was observed from all the hypnotics and placebo (*P* < 0.01) (Tables S2 and Figure S4). However, the subjective index measurements from the hypnotics were below the significant level.

## 4 DISCUSSION

In the present study, to enhance the assay sensitivity for residual effects, forced awakening at 4:00 was targeted when the elderly subjects tend to go to rest room. After waking up, all subjects maintained a wakeful state to complete the tests (4:00–4:30).

During first period, suvorexant had short REM latency and long REM sleep time, as previously reported (*P* = 0.049, *P* = 0.001, respectively, Table 2; Figure 3), which suggested that the mechanism of action is vastly contributed by orexin antagonism.^37^ For the N3 NREM sleep during first sleep period, the main effects of medicines were found (*P* = 0.006); however, significant differences were not found between each compound, ramelteon had shortest N3 sleep time (Table 2; Figure S1). Ramelteon is a melatonin receptor agonist that acts on the melatonin receptors, and regulates sleep and circadian rhythm; thus, continuous administration is reported to be more effective. Therefore, a single administration as in this study would have weak effect on sleep, especially stage3 NREM sleep.^13^ The time in bed, TST, SL, and sleep efficiency were not significantly different among all the conditions from 23:00 to 4:00 (Table 2).

During second period, the main effects of medicines were seen for TST (*P* = 0.039, Table 3). The main effects of medicines were seen for SL and N2 sleep time (*P* = 0.011, *P* = 0.035). SL of suvorexant was significantly shorter than that of ramelteon with post hoc test (*P* = 0.036, Table 3; Figure 2B; Figure 4). However, there were no significant differences between each compound in N2 sleep time (Table 3).

A significant difference was observed in REM sleep time between suvorexant and zolpidem. It could be speculated that zolpidem suppresses REM sleep while suvorexant increases REM sleep.^16^, ^37^, ^38^ Orexin receptor antagonists establish a sleep‐permissive state in insomniac patients by specifically blocking the wake‐promoting effects of orexin peptides.^16^, ^39^, ^40^ In contrast, GABAergic agonists such as zolpidem increase neuronal inhibitory tone, promoting sleep but also impacting neurophysiological activity in a widespread, non‐specific manner throughout the central nervous system.^41^ Among the GABAergic agonists currently prescribed, zolpidem was selected as a positive control because this compound is one of the most prescribed hypnotics in Japan.^42^, ^43^

When the subjects were allowed to go back to sleep at 4:30, suvorexant had shorter SL than ramelteon, since the half‐life of suvorexant (12 h) was longer than that of ramelteon (1‐2 h; Figure 2A).^43^ Although it is not clear whether short SL has a positive correlation with longer N2 sleep, it could be speculated that since the subjects fell asleep in short duration, it was possible for a longer N2 sleep time under the influence of suvorexant (Figure 2).

In the objective measurements, body sway movements with their eyes closed after zolpidem administration were significantly less than after suvorexant and ramelteon administrations (Table 4; Figure 5). Other parameters of subjective and objective measurements were not significantly different (Table S2; Figures S4).

It has been pointed out that there is a relationship between hypnotics and motor and cognitive dysfunctions of the elderly.^4^, ^5^, ^6^, ^7^, ^8^ The present study examined the safety of low dose of hypnotics from early morning to afternoon on the next day when the side effects tend to occur. Not only the previous studies reported that low doses were effective in improving total sleep time and sleep latencies, but also they are currently recommended dosage by physicians.^44^, ^45^, ^46^, ^47^, ^48^ It was assumed that the physical‐ and cognitive‐function tasks were performed under the influence of the hypnotic agents; especially at 4:00 and 6:00, when main effects were observed in TUG (*P* = 0.002), Body sway test; eyes open (P=0.002), CFF (*P* = 0.0001), SDR‐time (*P* = 0.01) and STM (*P* = 0.02) examinations without variance among the medications (Table 4; Figure 5). There were no serious adverse events. Therefore, it is considered that these three drugs are not problematic for the elderly to take a single dose administration.

Previous reports of hypnotics and falls have reported that suvorexant and ramelteon are safer than GABAa hypnotics, but they are also reported to be more dangerous, and thus, are inconsistent.^14^, ^49^, ^50^ From the clinical data of the real world, the inconsistencies were reported by Ishigo et al.^50^ that suvorexant and ramelteon were better than GABAa hypnotics for prevention of falls from their case‐crossover study. Both case‐control and case‐crossover studies by Torii et al.^14^ addressed those patients performed better under the influence of suvorexant than of ramelteon or GABAa hypnotics; whereas Ishibashi et al.^49^ reported that ramelteon should be more recommended than suvorexant or GABAa hypnotics through a case‐crossover study.

There were no significant drug differences in the dynamic balance parameters, FRT, and TUG, which are the optimal indices of falls.^33^, ^34^ On the contrary, zolpidem had significantly less movement than ramelteon and suvorexant in body sway test with closed eyes, which is often used in the side effect evaluation of medicines. Since there was no difference between the three drugs in the dynamic balance, it is unclear why there was a significant difference only in the eyes closed static balance. Similar results were seen in the previous study of Sol et al.^37^ Suvorexant exerts a therapeutic effect in insomnia through antagonism at the orexin receptors. Unlike benzodiazepines and non‐benzodiazepines, suvorexant and ramelteon do not affect the GABA system, so they have little muscle relaxant effect and respiratory depression are considered to be relatively safe for elderly people.^51^, ^52^, ^53^

Although it has been reported that suvorexant carried over at 15 mg^54^, ^55^ there was no carry‐over for the 10 mg that we used in this study.^56^ Considering the subjective indexes, the main effect of time was observed in all hypnotics, but the main effects of the hypnotics and placebo were not significantly different.

As mentioned above, the subjects were administered with a low single dosage of each medication in between a week of washout period. Even though we administered only once with low dosage, we cannot rule out the possibility that there could be interaction of kinetics of different active agents affecting our test results.

Based on the above results, the changes of physical and cognitive functions in healthy elderly after taking hypnotics in this single low dosage administration were not remarkable during the night after the administrations and its following day.

### 4.1 Limitations

The next‐day residual effects of the commercially available hypnotics in Japan, following a bedtime single dosing in healthy elderly subjects, were evaluated. Since we conducted this study with a low, single dosage, we need to take into account of the lack of dose response. The influences of continuous hypnotic use also need to be considered. Since elderly patients are quite often administered with long‐term hypnotics; of some are prescribed with multiple medications, further studies should be investigated. We performed the tests on highly selective and few relatively young elderly subjects without any insomnia; thus, there should be further investigations comparing low doses of zolpidem, suvorexant, and ramelteon on older elderly subjects with insomnia. The setting we used was not home based, but in a hotel, which should also be considered as a caveat.

## 5 CONCLUSION

From these results, no adverse side effects were observed with a single dose of the zolpidem, suvorexant, and ramelteon in the elderly. Suvorexant had significantly longer REM sleep than zolpidem. Zolpidem had significantly less movement than ramelteon and suvorexant in body sway test with eyes closed. Ramelteon did not show clear differences from other compounds. Therefore, these three hypnotics of single low dosage administration would not be a risk for the elderly people with insomnia. Physicians should be advised to recommend the patients with proper guidelines which introduce merits and demerits of each medication not only for sleep but also for activity during the following day.

## AUTHOR CONTRIBUTIONS

Sachiko Ito Uemura, Takashi Kanbayashi and Seiji Nishino is Conceived and designed the experiments; Sachiko Ito Uemura, Aya Imanishi, Yoshino Terui, Masahiro Satakea, and Takashi Kanbayashi is Performed the experiments; Sachiko Ito Uemura, Takashi Kanbayashi, and Seiji Nishino is Analyzed the data; Takanobu Shioyad, Takashi Kanbayashi, Insung Park, and GoEun Hanc is Contributed reagents/materials/analysis tools; Sachiko Ito Uemura, Takashi Kanbayashi and Seiji Nishino is Wrote the paper.

## CONFLICT OF INTEREST

The ICMJE Uniform Disclosure Form for Potential Conflicts of Interest associated with this article can be viewed by clicking on the following link: https://doi.org/10.1016/j.sleep.2015.05.021. Takashi Kanbayashi has received research grants from Eisai and speaker’s honoraria from Otsuka Pharmaceutical, MSD, Takeda, Sumitomo Dainippon Pharma and Eisai. Other authors do not have conflict of interest.

## APPROVAL OF THE RESEARCH PROTOCOL

This study was approved by Akita University Ethics Committee (No.1580).

## INFORMED CONSENT

This study was carried out in accordance with the principles based on the Declaration of Helsinki, and written informed consent was obtained from all subjects.

## REGISTRATION NO. OF THE STUDY

This study is registered in University hospital Medical Information Network (UMIN:R000053801).

## ANIMAL STUDIES

This study was strictly clinical; hence, there were no animal studies.

## Supporting information


Data S1

## Data Availability

The data that support the findings of this study are available on request from the corresponding author. The data are not publicly available due to privacy and ethical restrictions. The IRB did not grant the deposit of raw data in a publicly accessible data archive or repository at the time of approval since the procedure was not included in the study protocol or informed consent document.
