# Supplementary material for: Residual effects of low dose of suvorexant, zolpidem, and ramelteon in healthy elderly subjects: A randomized double‐blind study
Source: Neuropsychopharmacol Rep. 2022 Jun 24;42(3):288–98. doi: 10.1002/npr2.12262 (PMC9515713; doi:10.1002/npr2.12262)
Supplement: Supplementary file 1 — Data S1 [file NPR2-42-288-s001.pptx]

## Slide 1
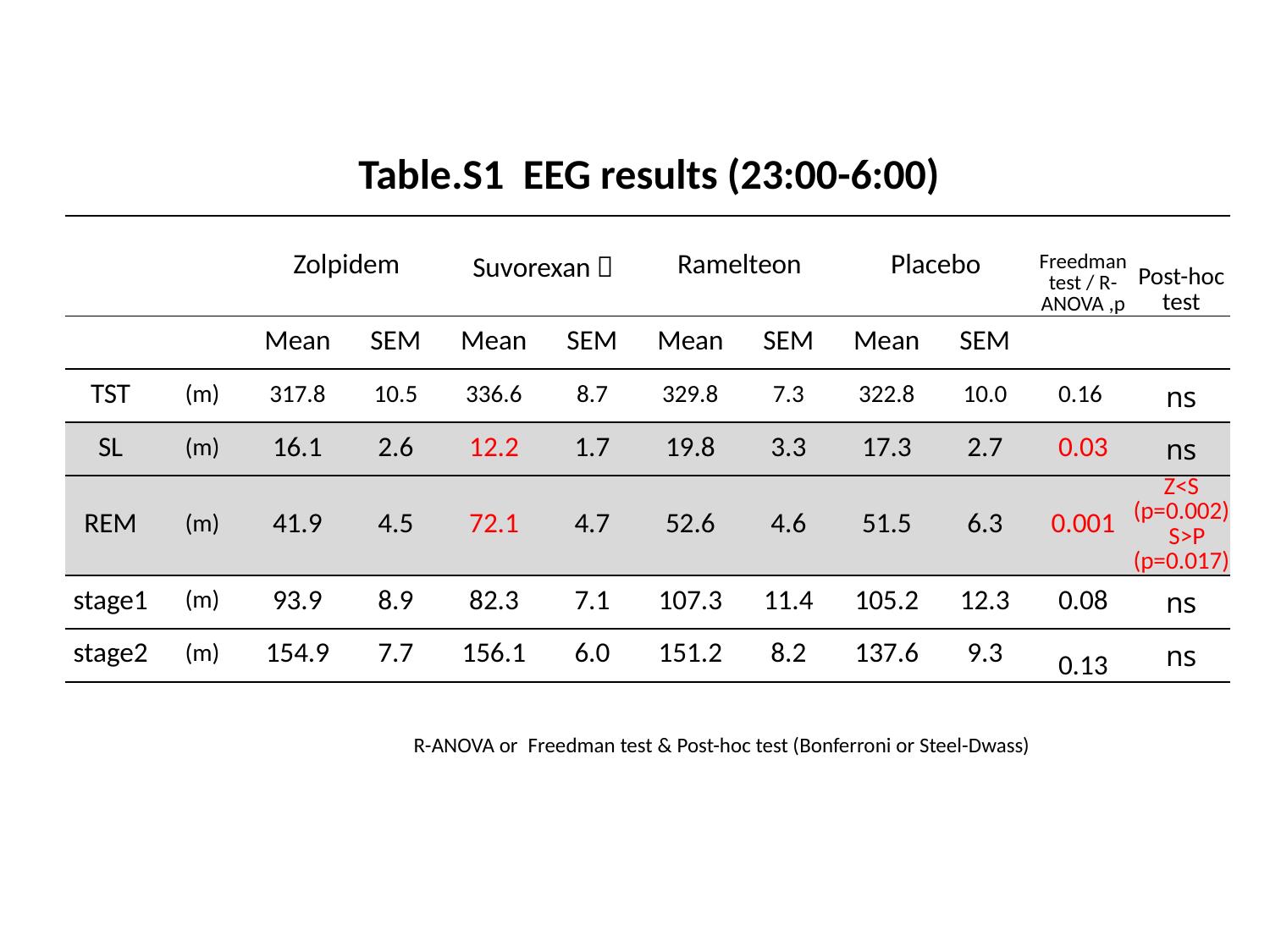

Table.S1 EEG results (23:00-6:00)
| | | Zolpidem | | Suvorexanｔ | | Ramelteon | | Placebo | | Freedman test / R-ANOVA ,p | Post-hoc test |
| --- | --- | --- | --- | --- | --- | --- | --- | --- | --- | --- | --- |
| | | Mean | SEM | Mean | SEM | Mean | SEM | Mean | SEM | | |
| TST | (m) | 317.8 | 10.5 | 336.6 | 8.7 | 329.8 | 7.3 | 322.8 | 10.0 | 0.16 | ns |
| SL | (m) | 16.1 | 2.6 | 12.2 | 1.7 | 19.8 | 3.3 | 17.3 | 2.7 | 0.03 | ns |
| REM | (m) | 41.9 | 4.5 | 72.1 | 4.7 | 52.6 | 4.6 | 51.5 | 6.3 | 0.001 | Z<S (p=0.002) S>P (p=0.017) |
| stage1 | (m) | 93.9 | 8.9 | 82.3 | 7.1 | 107.3 | 11.4 | 105.2 | 12.3 | 0.08 | ns |
| stage2 | (m) | 154.9 | 7.7 | 156.1 | 6.0 | 151.2 | 8.2 | 137.6 | 9.3 | 0.13 | ns |
　R-ANOVA or Freedman test & Post-hoc test (Bonferroni or Steel-Dwass)

## Slide 2
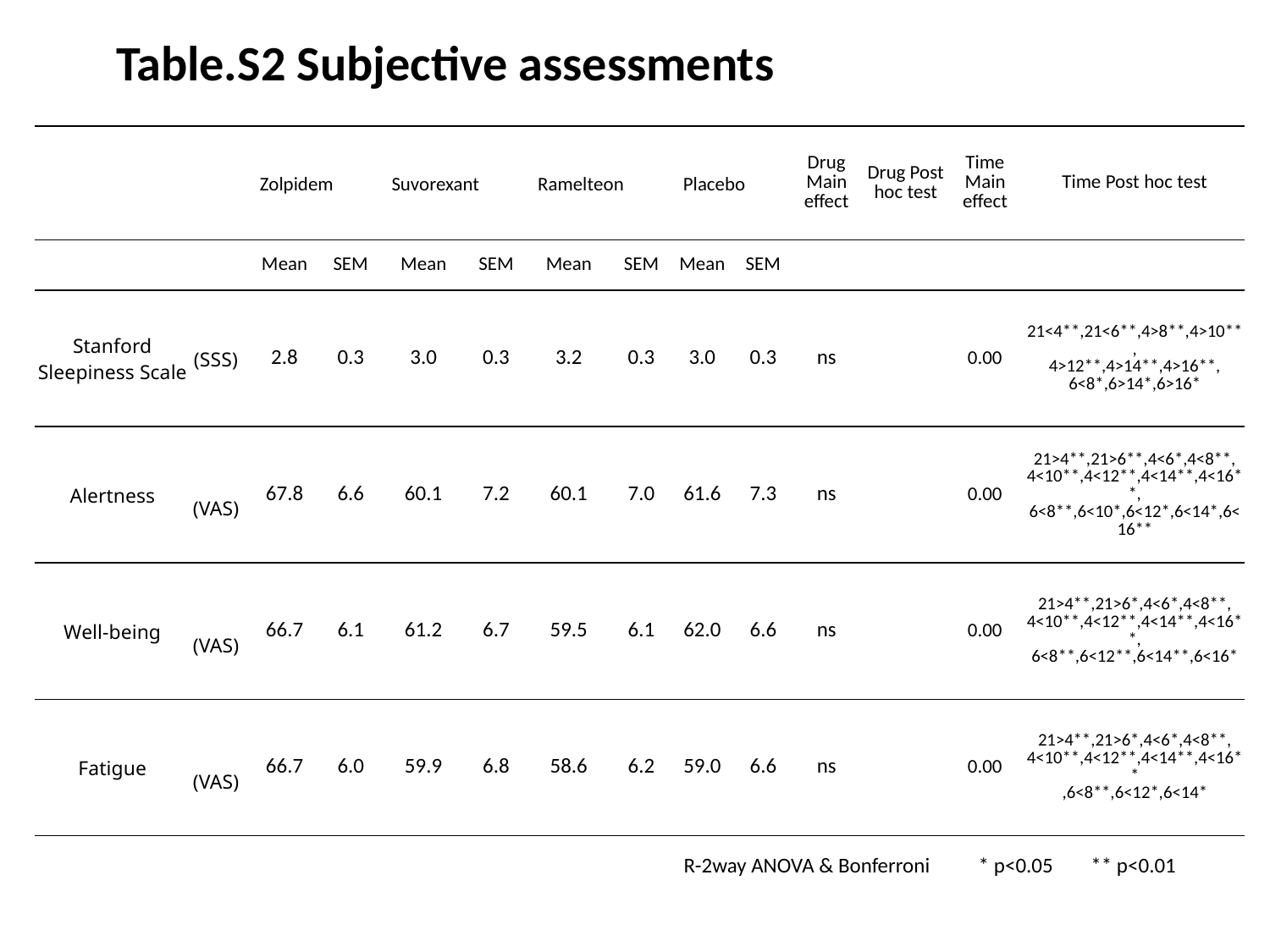

Table.S2 Subjective assessments
| | | Zolpidem | | Suvorexant | | Ramelteon | | Placebo | | Drug Main effect | Drug Post hoc test | Time Main effect | Time Post hoc test |
| --- | --- | --- | --- | --- | --- | --- | --- | --- | --- | --- | --- | --- | --- |
| | | Mean | SEM | Mean | SEM | Mean | SEM | Mean | SEM | | | | |
| Stanford Sleepiness Scale | (SSS) | 2.8 | 0.3 | 3.0 | 0.3 | 3.2 | 0.3 | 3.0 | 0.3 | ns | | 0.00 | 21<4\*\*,21<6\*\*,4>8\*\*,4>10\*\*, 4>12\*\*,4>14\*\*,4>16\*\*, 6<8\*,6>14\*,6>16\* |
| Alertness | (VAS) | 67.8 | 6.6 | 60.1 | 7.2 | 60.1 | 7.0 | 61.6 | 7.3 | ns | | 0.00 | 21>4\*\*,21>6\*\*,4<6\*,4<8\*\*, 4<10\*\*,4<12\*\*,4<14\*\*,4<16\*\*, 6<8\*\*,6<10\*,6<12\*,6<14\*,6<16\*\* |
| Well-being | (VAS) | 66.7 | 6.1 | 61.2 | 6.7 | 59.5 | 6.1 | 62.0 | 6.6 | ns | | 0.00 | 21>4\*\*,21>6\*,4<6\*,4<8\*\*, 4<10\*\*,4<12\*\*,4<14\*\*,4<16\*\*, 6<8\*\*,6<12\*\*,6<14\*\*,6<16\* |
| Fatigue | (VAS) | 66.7 | 6.0 | 59.9 | 6.8 | 58.6 | 6.2 | 59.0 | 6.6 | ns | | 0.00 | 21>4\*\*,21>6\*,4<6\*,4<8\*\*, 4<10\*\*,4<12\*\*,4<14\*\*,4<16\*\* ,6<8\*\*,6<12\*,6<14\* |
　R-2way ANOVA & Bonferroni * p<0.05　 ** p<0.01

## Slide 3
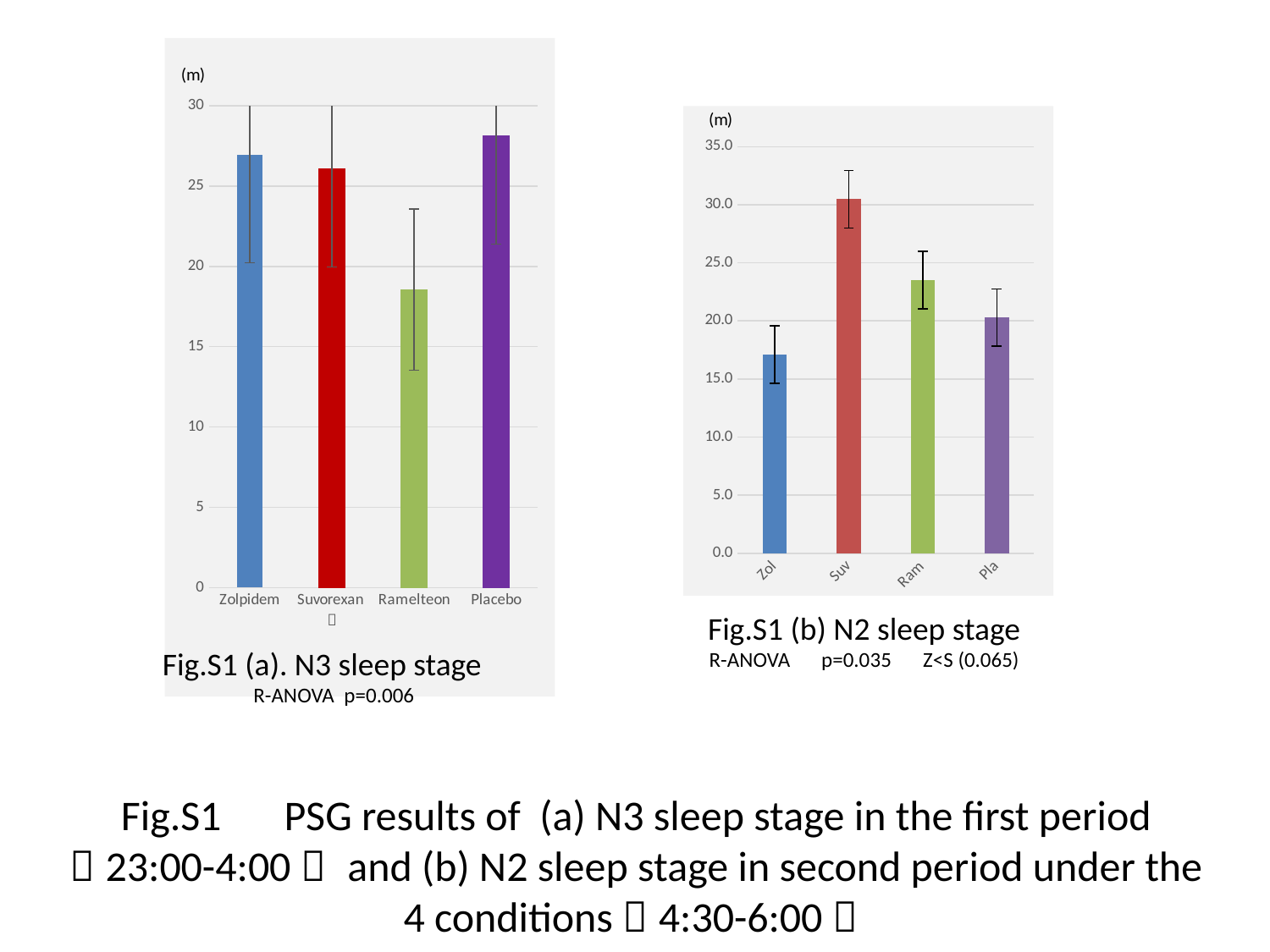

### Chart
| Category | |
|---|---|
| Zolpidem | 26.96428571428572 |
| Suvorexanｔ | 26.07142857142857 |
| Ramelteon | 18.55714285714285 |
| Placebo | 28.10714285714285 |(m)
### Chart
| Category | |
|---|---|
| Zolpidem | 17.10714285714285 |
| Suvorexanｔ | 30.46153846153846 |
| Ramelteon | 23.5 |
| Placebo | 20.28571428571408 |Fig.S1 (b) N2 sleep stage
R-ANOVA　p=0.035　Z<S (0.065)
Fig.S1 (a). N3 sleep stage
R-ANOVA p=0.006
Fig.3-3. Stage 3
Fig.S1　PSG results of (a) N3 sleep stage in the first period （23:00-4:00） and (b) N2 sleep stage in second period under the 4 conditions（4:30-6:00）

## Slide 4
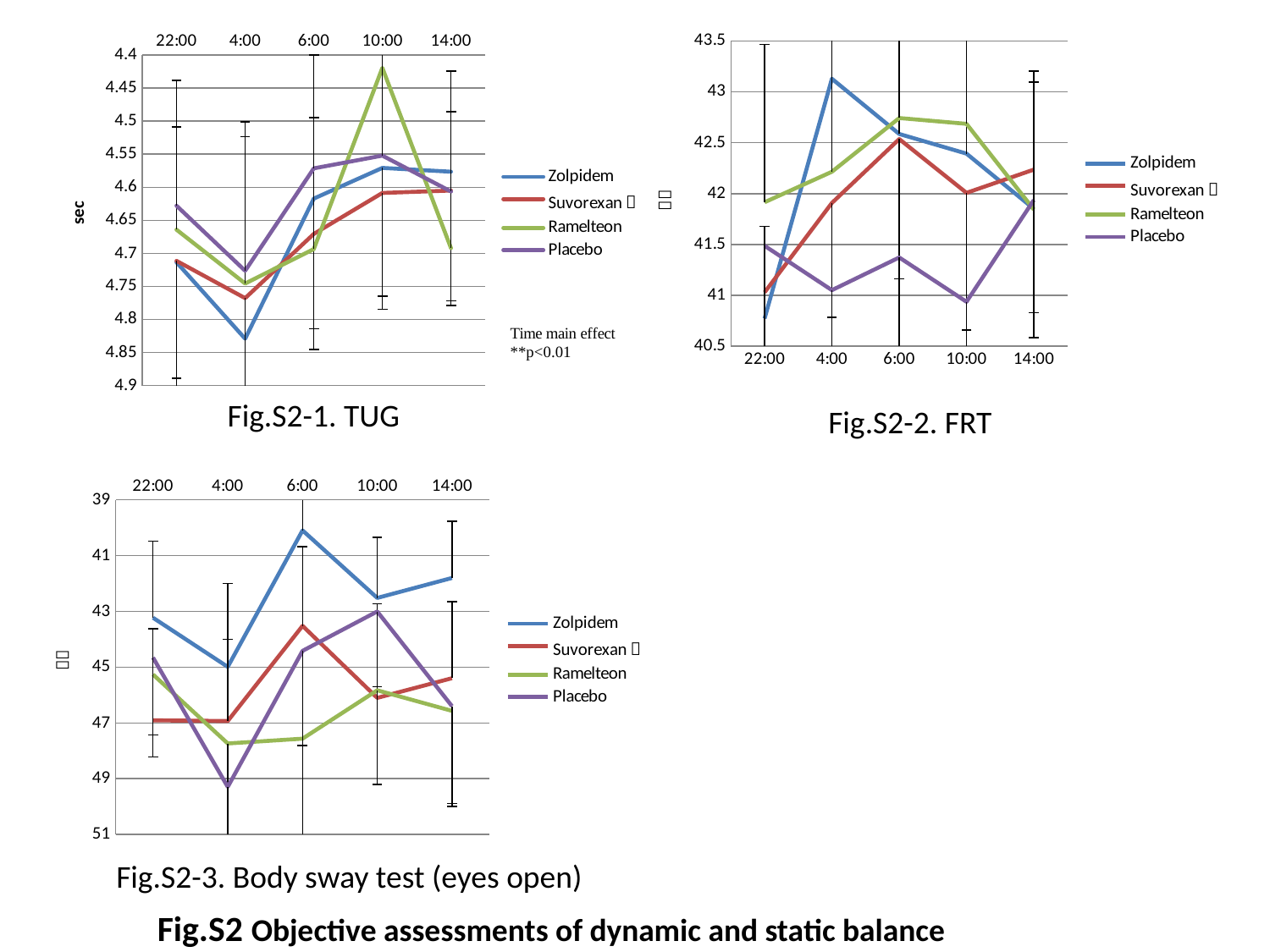

### Chart
| Category | Zolpidem | Suvorexanｔ | Ramelteon | Placebo |
|---|---|---|---|---|
| 0.91666666666666596 | 4.713571428571428 | 4.711428571428571 | 4.664285714285675 | 4.627857142857135 |
| 0.16666666666666699 | 4.829285714285715 | 4.76785714285714 | 4.745714285714286 | 4.72642857142857 |
| 0.25 | 4.617142857142824 | 4.670714285714285 | 4.693571428571428 | 4.571428571428571 |
| 0.41666666666666702 | 4.570714285714286 | 4.608571428571428 | 4.419285714285715 | 4.552142857142845 |
| 0.58333333333333304 | 4.576428571428571 | 4.604999999999975 | 4.692142857142834 | 4.606428571428569 |
### Chart
| Category | Zolpidem | Suvorexanｔ | Ramelteon | Placebo |
|---|---|---|---|---|
| 0.91666666666666596 | 40.77142857142852 | 41.02857142857143 | 41.91428571428528 | 41.48571428571427 |
| 0.16666666666666699 | 43.12857142857146 | 41.90714285714282 | 42.21428571428572 | 41.05 |
| 0.25 | 42.58571428571425 | 42.53571428571428 | 42.74285714285715 | 41.37142857142812 |
| 0.41666666666666702 | 42.39285714285715 | 42.00714285714285 | 42.6857142857143 | 40.9357142857143 |
| 0.58333333333333304 | 41.85 | 42.23571428571428 | 41.84285714285714 | 41.9357142857143 |Fig.S2-1. TUG
Fig.S2-2. FRT
### Chart
| Category | Zolpidem | Suvorexanｔ | Ramelteon | Placebo |
|---|---|---|---|---|
| 0.91666666666666596 | 43.2269632307143 | 46.90553909785715 | 45.26409088428572 | 44.65407885571383 |
| 0.16666666666666699 | 44.98853121071428 | 46.93179043428571 | 47.73463694642857 | 49.29284681714285 |
| 0.25 | 40.0963031857143 | 43.5183059607143 | 47.56394325214286 | 44.41502745499999 |
| 0.41666666666666702 | 42.51707954285715 | 46.10113539714286 | 45.82852145714227 | 43.00275091357143 |
| 0.58333333333333304 | 41.80053095928572 | 45.39649950571382 | 46.56737252357143 | 46.39823346 |Fig.S2-3. Body sway test (eyes open)
Fig.S2 Objective assessments of dynamic and static balance

## Slide 5
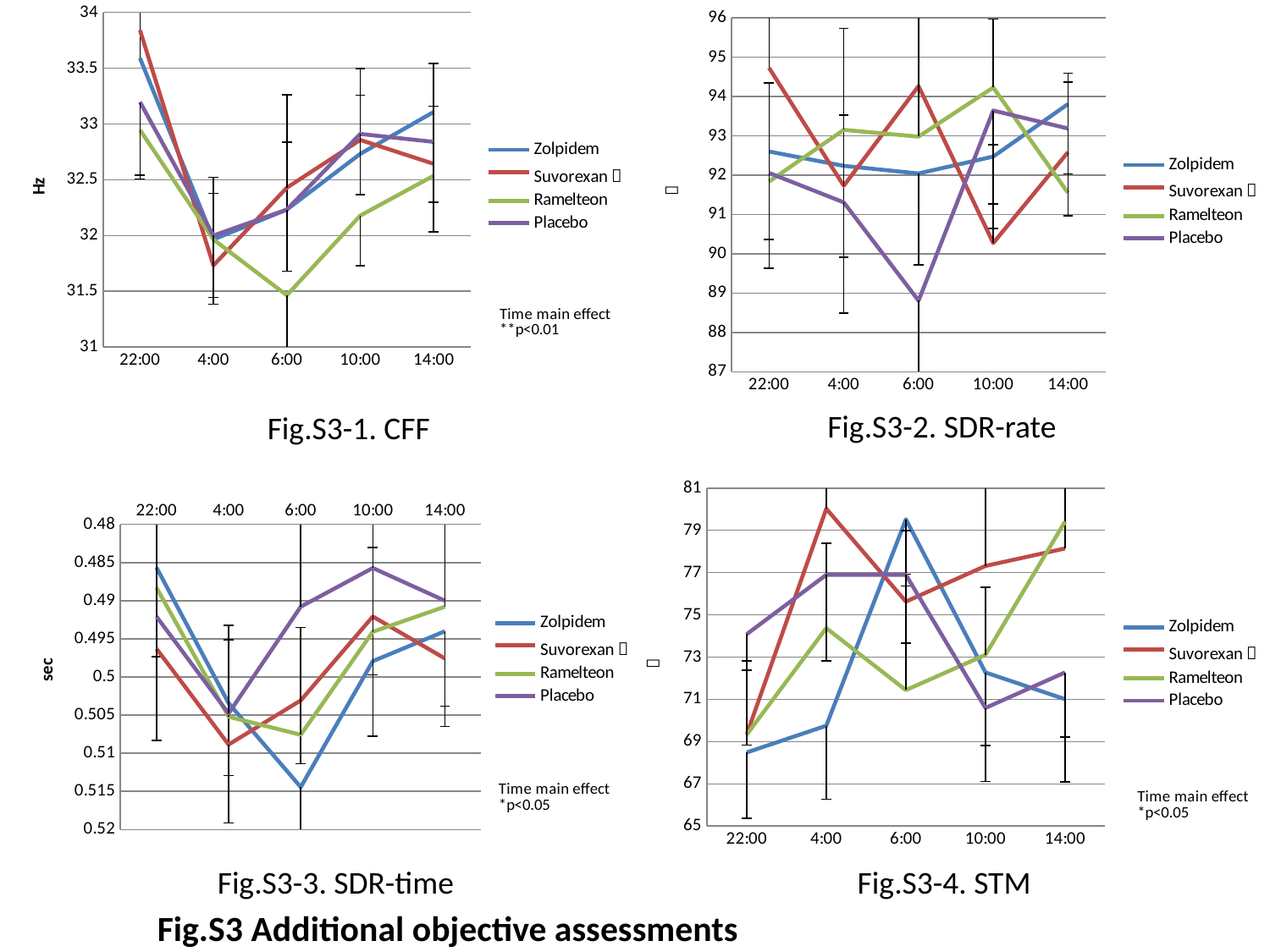

### Chart
| Category | Zolpidem | Suvorexanｔ | Ramelteon | Placebo |
|---|---|---|---|---|
| 0.91666666666666596 | 33.58928571428572 | 33.83928571428572 | 32.94642857142829 | 33.19642857142856 |
| 0.16666666666666699 | 31.96428571428572 | 31.73214285714278 | 31.96428571428572 | 32.0 |
| 0.25 | 32.23214285714286 | 32.42857142857143 | 31.46428571428572 | 32.23214285714286 |
| 0.41666666666666702 | 32.73214285714286 | 32.85714285714226 | 32.17857142857143 | 32.91071428571419 |
| 0.58333333333333304 | 33.10714285714285 | 32.64285714285715 | 32.53571428571428 | 32.83928571428572 |
### Chart
| Category | Zolpidem | Suvorexanｔ | Ramelteon | Placebo |
|---|---|---|---|---|
| 0.91666666666666596 | 92.60000000000001 | 94.72857142857085 | 91.8357142857143 | 92.05714285714285 |
| 0.16666666666666699 | 92.2357142857143 | 91.72857142857085 | 93.15 | 91.30714285714285 |
| 0.25 | 92.04285714285626 | 94.2642857142857 | 92.97857142857085 | 88.80714285714285 |
| 0.41666666666666702 | 92.47142857142845 | 90.27142857142825 | 94.22857142857085 | 93.64285714285604 |
| 0.58333333333333304 | 93.80714285714285 | 92.5857142857143 | 91.55 | 93.18571428571428 |Fig.S3-2. SDR-rate
Fig.S3-1. CFF
### Chart
| Category | Zolpidem | Suvorexanｔ | Ramelteon | Placebo |
|---|---|---|---|---|
| 0.91666666666666596 | 68.4857142857143 | 69.3357142857143 | 69.3285714285707 | 74.0857142857143 |
| 0.16666666666666699 | 69.75000000000001 | 80.02142857142825 | 74.37142857142794 | 76.89285714285604 |
| 0.25 | 79.52142857142825 | 75.62857142857048 | 71.4357142857143 | 76.89285714285604 |
| 0.41666666666666702 | 72.27142857142825 | 77.31428571428572 | 73.10714285714275 | 70.59285714285616 |
| 0.58333333333333304 | 71.00714285714285 | 78.14285714285604 | 79.41428571428573 | 72.27142857142825 |
### Chart
| Category | Zolpidem | Suvorexanｔ | Ramelteon | Placebo |
|---|---|---|---|---|
| 0.91666666666666596 | 0.485642857142857 | 0.496285714285714 | 0.488285714285714 | 0.492071428571429 |
| 0.16666666666666699 | 0.503428571428572 | 0.508857142857143 | 0.505214285714286 | 0.504785714285714 |
| 0.25 | 0.514428571428572 | 0.503071428571429 | 0.507571428571429 | 0.490785714285714 |
| 0.41666666666666702 | 0.497928571428571 | 0.492071428571428 | 0.494071428571429 | 0.485714285714286 |
| 0.58333333333333304 | 0.494 | 0.497571428571429 | 0.490785714285714 | 0.49 |Fig.S3-4. STM
Fig.S3-3. SDR-time
Fig.S3 Additional objective assessments

## Slide 6
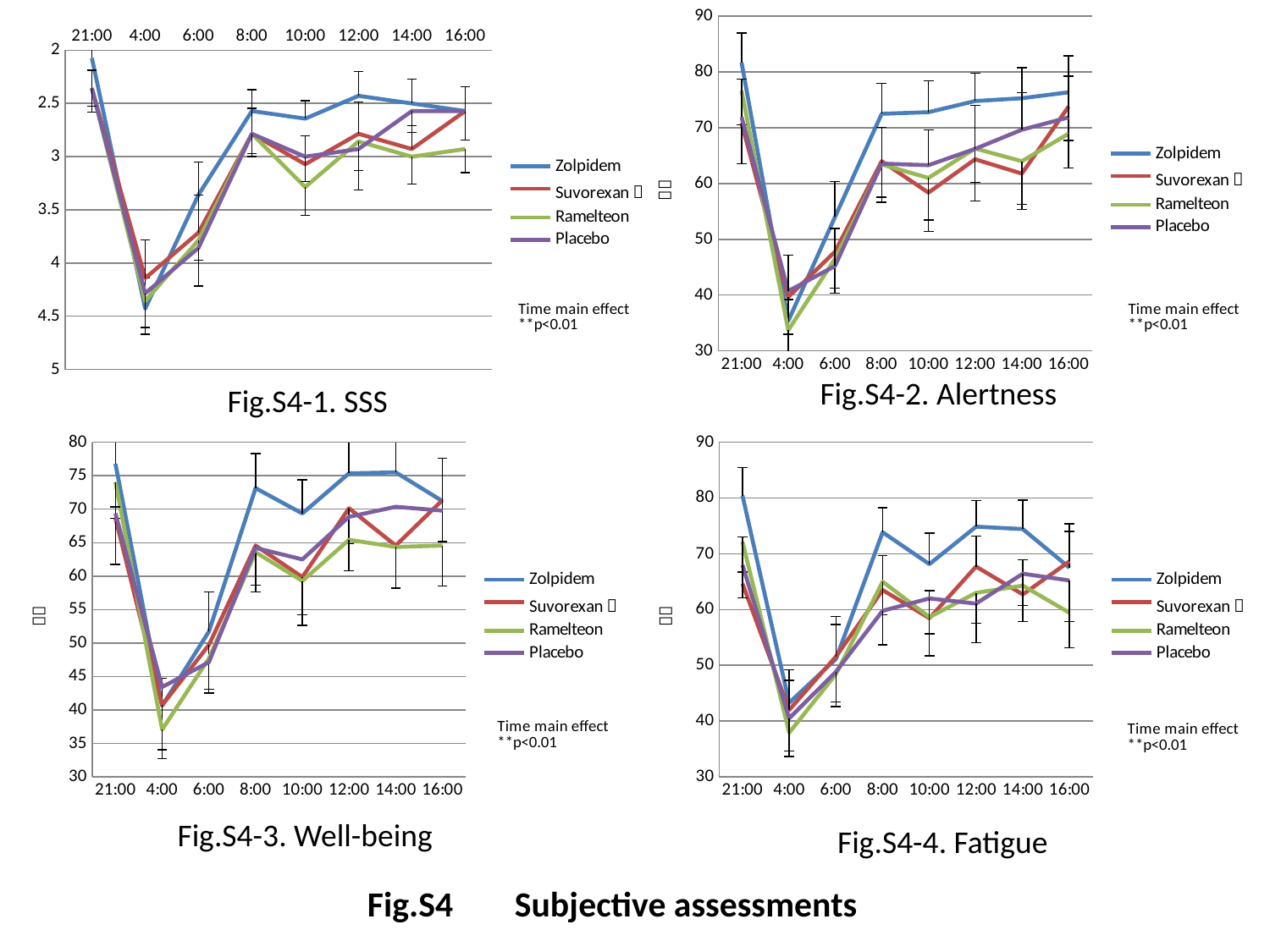

### Chart
| Category | Zolpidem | Suvorexanｔ | Ramelteon | Placebo |
|---|---|---|---|---|
| 0.875 | 81.71428571428572 | 70.85714285714275 | 76.64285714285604 | 71.92857142857125 |
| 0.16666666666666699 | 35.35714285714226 | 39.64285714285715 | 33.71428571428572 | 40.71428571428572 |
| 0.25 | 54.0 | 47.78571428571428 | 46.5 | 45.21428571428572 |
| 0.33333333333333298 | 72.5 | 64.0 | 63.42857142857143 | 63.57142857142829 |
| 0.41666666666666702 | 72.7857142857143 | 58.35714285714226 | 61.0 | 63.28571428571428 |
| 0.5 | 74.7857142857143 | 64.35714285714275 | 66.2857142857143 | 66.21428571428572 |
| 0.58333333333333304 | 75.2857142857143 | 61.78571428571428 | 64.0 | 69.64285714285604 |
| 0.66666666666666696 | 76.35714285714275 | 73.7857142857143 | 68.92857142857125 | 71.85714285714275 |
### Chart
| Category | Zolpidem | Suvorexanｔ | Ramelteon | Placebo |
|---|---|---|---|---|
| 0.875 | 2.071428571428571 | 2.357142857142857 | 2.357142857142857 | 2.357142857142857 |
| 0.16666666666666699 | 4.428571428571429 | 4.142857142857141 | 4.357142857142835 | 4.285714285714286 |
| 0.25 | 3.357142857142857 | 3.714285714285714 | 3.785714285714286 | 3.857142857142857 |
| 0.33333333333333298 | 2.571428571428571 | 2.785714285714286 | 2.785714285714286 | 2.785714285714286 |
| 0.41666666666666702 | 2.642857142857143 | 3.071428571428571 | 3.285714285714286 | 3.0 |
| 0.5 | 2.42857142857143 | 2.785714285714286 | 2.857142857142857 | 2.92857142857143 |
| 0.58333333333333304 | 2.5 | 2.92857142857143 | 3.0 | 2.571428571428571 |
| 0.66666666666666696 | 2.571428571428571 | 2.571428571428571 | 2.92857142857143 | 2.571428571428571 |Fig.S4-2. Alertness
Fig.S4-1. SSS
### Chart
| Category | Zolpidem | Suvorexanｔ | Ramelteon | Placebo |
|---|---|---|---|---|
| 0.875 | 76.7857142857143 | 68.64285714285604 | 74.07142857142794 | 69.35714285714275 |
| 0.16666666666666699 | 40.64285714285715 | 40.71428571428572 | 37.07142857142829 | 43.42857142857143 |
| 0.25 | 51.71428571428572 | 49.71428571428572 | 47.71428571428572 | 47.14285714285715 |
| 0.33333333333333298 | 73.14285714285604 | 64.57142857142794 | 63.57142857142829 | 64.21428571428572 |
| 0.41666666666666702 | 69.35714285714275 | 59.85714285714226 | 59.28571428571428 | 62.5 |
| 0.5 | 75.35714285714275 | 70.14285714285604 | 65.42857142857125 | 68.85714285714275 |
| 0.58333333333333304 | 75.5 | 64.57142857142794 | 64.35714285714275 | 70.35714285714275 |
| 0.66666666666666696 | 71.21428571428572 | 71.35714285714275 | 64.57142857142794 | 69.7857142857143 |
### Chart
| Category | Zolpidem | Suvorexanｔ | Ramelteon | Placebo |
|---|---|---|---|---|
| 0.875 | 80.42857142857125 | 64.64285714285604 | 72.14285714285604 | 68.0 |
| 0.16666666666666699 | 43.28571428571428 | 42.0 | 37.92857142857143 | 40.5 |
| 0.25 | 51.14285714285715 | 51.57142857142829 | 48.42857142857143 | 48.85714285714226 |
| 0.33333333333333298 | 73.85714285714275 | 63.5 | 65.0 | 59.78571428571428 |
| 0.41666666666666702 | 68.14285714285604 | 58.42857142857143 | 58.64285714285715 | 62.0 |
| 0.5 | 74.85714285714275 | 67.71428571428572 | 63.0 | 61.07142857142829 |
| 0.58333333333333304 | 74.42857142857125 | 62.71428571428572 | 64.2857142857143 | 66.42857142857125 |
| 0.66666666666666696 | 67.5 | 68.64285714285604 | 59.42857142857143 | 65.21428571428572 |Fig.S4-3. Well-being
Fig.S4-4. Fatigue
Fig.S4　 Subjective assessments
